# How problem difficulty and order influence programming education outcomes in online judge systems

**DOI:** 10.1016/j.heliyon.2023.e20947

**Published:** 2023-10-16

**Authors:** Jinshui Wang, Pengchen Lin, Zhengyi Tang, Shuguang Chen

**Affiliations:** a School of Computer Science and Mathematics, Fujian University of Technology, Fuzhou, 350100, Fujian, China; b Fujian Provincial Key Laboratory of Big Data Mining and Applications, Fuzhou, 350100, Fujian, China

**Keywords:** Online judge systems, Programming education, Problem difficulty, Behavior patterns, Cognitive load

## Abstract

With the widespread application of computer technology in engineering education, Online Judge (OJ) systems have become an important platform for programming teaching. OJ systems provide a platform for learners to practice programming skills, submit solutions, and receive feedback. They offer a conducive environment for learners to engage in hands-on coding exercises and enhance their programming abilities. This article explores the use of OJ systems as a software tool for enhancing programming education in engineering. It investigates how the difficulty and order of programming problems affect the users' behavior, performance, and cognitive load in OJ environments. The research data were sourced from Project_CodeNet. Using statistical methods, such as Spearman correlation analysis and differential analysis, the study reveals the factors that influence the users' submission situations, answer order, and learning outcomes. The findings provide useful implications for OJ system developers, teachers, and learners in designing, implementing, and using OJ systems for programming education in engineering. The study suggests that problem difficulty and order should be considered and adjusted according to the users' abilities and progress, to provide appropriate challenges and support, balance the cognitive load, and improve the programming skills of the users.

## 1 Introduction

Programming education is an essential part of engineering education, as it fosters students' computational thinking and problem-solving abilities [1]. However, students often face various challenges in learning programming, such as understanding abstract concepts and languages, mastering complex algorithms and data structures, and debugging errors. These challenges require students to overcome them through extensive practice. It is only through hands-on experience that students can truly grasp programming skills and develop a computational mindset [2]. By completing various programming exercises and course assignments, students can continuously enhance their understanding of programming knowledge and deepen their mastery of programming skills.

To facilitate students' learning of programming, the emergence of OJ (Online Judge) systems has been introduced. OJ are web-based learning platforms that provide engineering students with a variety of programming exercises and tasks related to various programming concepts, help them apply their theoretical knowledge to practical programming problems, and thereby enhance their programming skills and proficiency. Consequently, the OJ platform has become a popular way for individuals to learn programming worldwide. The primary method for students to use OJ is by submitting programming assignments online. When students submit their programming assignments, the OJ system automatically compiles, runs, and evaluates their submitted code against predefined test cases, and provide assessment results [3]. During the process of learning programming on the OJ platform, students may need to refine their solutions through multiple attempts and incorporate feedback provided by the OJ system. Within the OJ system, students' submission activities are recorded as submission information, including the submitted source code, CPU time and memory usage during code execution, and whether the code passes the test cases. Typically, students make multiple attempts on the same problem, and these attempts' submission records are not deleted or overwritten. As a result, students often keep multiple distinct submission records for a particular problem. Therefore, OJ systems provide a rich source of data for analyzing students' learning behavior and performance in programming education.

The difficulty level of problems on the OJ platform affects students' learning behavior and outcomes substantially. OJ platforms typically offer a wide range of programming problems, each assigned with its own difficulty level, such as easy, medium, and hard. In some cases, problem difficulty is classified more precisely using difficulty levels like 1 or difficulty coefficients like 0.95. For students learning programming through OJ, completing overly simple problems may lack challenge and fail to effectively enhance their programming skills. Conversely, attempting excessively difficult problems can lead to strong feelings of frustration, thereby diminishing the effectiveness of learning. They may even demotivate students and result in learning abandonment. The difficulty level of problems and the submission information related to those problems (such as the number of attempts and the number of individuals attempting the problem) serve as crucial means for educators to understand students' learning behavior and outcomes [4]. By investigating the influence of problem difficulty on submission patterns, educators can understand better how students handle problems of varying difficulty. Additionally, educators can assess students' learning progress and evaluate the effectiveness of teaching methods and course design by examining students' submission information and the submission patterns of problems. This provides a more scientific basis for improving teaching approaches and curriculum design. Therefore, it is important to understand how the difficulty level of problems affects students' learning behavior and outcomes on OJ platforms.

In addition to instructional OJ platforms, competitive OJ platforms such as Codeforces and AtCoder have also gained widespread popularity. For programming learners, participating in programming competitions offers a valuable learning experience and serves as a common way to test programming abilities and improve skill levels. During competition, users' solving problems may exhibit certain order characteristics. They may be influenced by the order of problem arrangement and consciously solve the problems in a structured manner. Alternatively, they may be influenced by the difficulty of problems and consciously avoid overly challenging ones, prioritizing relatively easier problems. In programming competition, there is no direct association between the difficulty level of problems and their sequential appearance. This means that adjacent problems may have a significant difference in difficulty, and more difficult problems may appear earlier than simpler ones. Consequently, this phenomenon gives rise to variations in the arrangement of problem difficulty in competitions. Easy problems and difficult problems present different levels of challenges, potentially leading to varying levels of pressure and test-taking experiences for participants. Therefore, when the order of difficulty varies, users' performance in competition may be influenced.

In competitive environments such as competitions and exams, the psychological factors of participants significantly influence their performance in answering problems [5]. Cognitive load is often considered as part of these psychological factors, involving an individual's attention, working memory load, and information processing demands during cognitive tasks. When participants experience higher cognitive load in a competition, they may require more cognitive resources, stronger cognitive abilities, and focused attention to handle the tasks, leading to increased pressure and a sense of challenge. Different difficulty levels of problems entail varying levels of complexity and require different cognitive resources, leading to differences in cognitive load. Therefore, when the order of problem difficulty varies, participants may face differences in cognitive load during the competition. Understanding the factors influencing participants' answer order and investigating the influence of different arrangements of problem difficulty on their performance can assist teachers and educators in organizing programming learning and assessments more effectively. By optimizing the order and difficulty setting of problems, a better learning and test-taking experience can be provided for learners. Additionally, this approach facilitates the implementation of personalized learning and teaching in programming education. Through appropriate arrangement of problem difficulty, teachers can balance students' challenges and pressures, thus fostering their enthusiasm to learn programming. To the best of our knowledge, few studies have investigated how the difficulty level and the order of programming problems on OJ platforms influence students' learning behavior, performance, and cognitive load. Therefore, this study aims to fill this research gap and provide insights for improving programming education and assessment. By analyzing the data collected from a competitive OJ platform, we explore the following research questions:

**RQ1.** How does the difficulty of a problem affect its submission situation? In particular:

• How does the difficulty of a problem affect the number of submissions it receives?

• How does the difficulty of a problem affect the number of people who submit it?

**RQ2.** What factors influence the answer order of users? Specifically, in the following aspects:

• How does the order of problems influence the answer order of users?

• How does the difficulty of problems influence the answer order of users?

**RQ3.** How does the difficulty arrangement order of problems affect users' answer outcomes?

**RQ4.** How does different difficulty arrangement order of problems lead to differences in cognitive load?

The rest of the article is organized as follows: section 2 provides an overview of related research in the field of computer-assisted instruction; section 3 introduces the dataset and data preprocessing methods used in this study, as well as the analytical approach to the research questions; section 4 presents the results of the data analysis and discusses these findings; section 5 summarizes the research results and proposes directions for future work.

## 2 Related works

This paper aims to explore how the difficulty level and the order of programming problems on OJ platforms affect students' learning behavior, performance, and cognitive load. In this section, we review the related works in the following aspects:

In the field of computer-aided instruction and programming education, there have been numerous studies on educational data analysis and learning behavior patterns. Restrepo et al. [6] proposed a continuous assessment method for computer programming courses supported by software tools, that helps teachers understand students' learning process through the analysis of their learning data. Kokoç et al. [7] analyzed students' online homework submission patterns and identified factors affecting students' behavior patterns. These factors include homework deadlines, difficulty levels, and assignment types, all of which have an influence on students' submission behaviors. Sun et al. [8] used temporal learning analysis to examine the relationship between students' self-regulated learning behaviors and their course performance, cognitive load, and engagement. Berssanette et al. [9] evaluated and summarized the research and application of cognitive load theory in the field of computer programming education over the past two decades, providing references for teachers and educators in related fields. Additionally, many studies have focused on the relationship between students' online learning behaviors and their academic performance, such as Zhao et al. [10] and Akçapınar et al. [11].

In the application of educational data analysis, automated assessment and feedback are important research areas, and automated programming assignment assessment is a key focus. Caiza and Del Álamo Ramiro [12] provided a survey of automated programming assignment assessment tools and implementation methods, while Ihantola et al. [13] surveyed systems for automated assessment of programming assignments in recent years. Furthermore, Paule-Ruiz et al. [14] investigated indicators of procrastination behavior among students in e-learning platforms, while Zacharis [15] used multivariate analysis methods to predict student learning outcomes in online blended learning courses. Juhaňák et al. [16] employed process mining techniques to analyze students' behavior patterns during test participation in learning management systems. Al-Nasa'h et al. [17] conducted a cross-sectional survey from June 2020 to August 2020 to investigate the impact of self-efficacy, generalized anxiety, and COVID-19 fear on different levels of online learning satisfaction among university students. Cerezo et al. [18] explored personalized learning path recommendation methods based on students' knowledge states from the perspective of knowledge graphs, and provide personalized learning suggestions and guidance for students.

From the comprehensive review of the extant literature, it becomes apparent that while there have been myriad investigations into OJ systems and programming education, certain research lacunae persist. Current studies indicate that assignment difficulty affects students' submission behavior patterns [7]. To the best of our knowledge, there are few studies on the impact of problem difficulty on the submission situation in the context of programming competitions. This study explores the influence of programming problem difficulty on submission behavior, thus extending the existing research in this field. In the field of computer programming education, the research and application of cognitive load theory have achieved certain results. Investigating the factors that influence users' answer sequence contributes to understanding their answering habits. Building upon this, studying the influence of the difficulty ordering of problems on users' answer results and cognitive load can assist teachers and educational practitioners in designing learning processes and organizing programming assessments, thereby providing students with a better learning and examination experience. Therefore, this study aims to fill this research gap and provide insights for improving programming education and assessment. By analyzing the data collected from competitive OJ platforms, we explore how the difficulty level and the order of programming problems affect students' learning behavior, performance, and cognitive load. This study can help teachers and educators understand students' learning behavior and outcomes better, optimize the design and arrangement of programming problems, and provide personalized learning and teaching suggestions for students.

## 3 Data and methods

### 3.1 Experimental design

In this study, four research questions are proposed. In RQ1, to investigate the influence of problem difficulty on submission situation, the data are planned to be grouped based on difficulty levels. All problems will be categorized into four groups for subsequent analysis. During the analysis, descriptive statistics will be performed on the data and, based on its characteristics, appropriate correlational analysis methods will be selected for exploration.

In RQ2, to investigate the factors influencing answer sequence of users, 514,128 competition records are planned to be processed according to the definition of orderliness of the sequence (as detailed in Section 3.5). This process will ascertain the orderliness of each competition record for subsequent analysis. During the analysis phase, descriptive statistics will be performed on the data. Depending on the characteristics of the data, appropriate statistical methods will be selected for statistical test.

In RQ3, to investigate the impact of problem difficulty arrangement order on users' answer outcomes, competitions are planned to be categorized into three types based on their difficulty arrangement: “easy to difficult,” “difficult to easy,” and “no characteristic” (as detailed in Section 3.5). Additionally, users will be grouped according to their ability levels. Compared to ungrouped analysis, this approach can uncover differences in users at different ability levels when facing different difficulty arrangement order (as detailed in Section 4.3.1). During the analysis phase, the Mann-Whitney U test will be employed to assess the differences in answer outcomes for users of varying ability levels under different problem difficulty arrangement order.

In RQ4, for examining the disparities in cognitive load under different difficulty arrangement order, competitions will be categorized and users grouped in a manner consistent with research question 3. Subsequently, cognitive load is planned to be evaluated based on the Project_CodeNet dataset (as detailed in Section 4.4.2). During the analysis phase, the Mann-Whitney U test will be utilized to analyze differences in cognitive load among users of different ability levels across the various problem difficulty arrangement order.

### 3.2 Data source

In this study, we used IBM's publicly available Project_CodeNet dataset (https://github.com/IBM/Project_CodeNet) [19]. The dataset collected submission data from two online coding platforms, AIZU Online Judge and AtCoder. In Project_CodeNet, the primary data consists of user submission records. Besides anonymized user IDs, the dataset does not encompass any descriptive information about the users, such as their geographical location or educational background. Project_CodeNet includes the following information: (1) Details of all the problems, such as problem ID, time and memory constraints, and problem source. (2) Detailed information on all the submissions, including user ID (user ID and similar sensitive information have been anonymized in the dataset), problem ID, CPU time, memory usage, submission status, and submission time. (3) Source code for all the submissions. Of the submissions 53.6% are accepted, 29.5% are marked as wrong answer and the remaining suffer from one of the possible rejection causes. This dataset is suitable for our study because it contains rich and detailed information on students' submission data, problem data, and source code, which can help us analyze students' learning behavior, performance, and cognitive load on OJ platforms.

### 3.3 Data preprocessing

In this study, the difficulty of the problems is a crucial piece of information. In the Project_CodeNet, the difficulty of the problems is represented by the “rating” in the dataset-level metadata. However, all the data entries corresponding to the “rating” label are empty, making it impossible to directly obtain the difficulty of the problems. Therefore, in this study, the Rasch model is employed to assess the difficulty of the problems (see section 3.3 for details).

To minimize the influence of confounding variables and ensure data quality, we carried out preprocessing on the dataset. Firstly, we excluded submission data for problems with fewer than 3 unique users, as a low number of participants challenges accurately assess the difficulty of the problems. Secondly, we removed submission data from the AIZU platform and kept only the data from the AtCoder platform. This decision was made because AtCoder competitions have time restrictions, allowing users to submit only within specified time frames. After the competition ends, no further submissions are allowed (users can only participate in virtual competitions, i.e., “Virtual Contests” at AtCoder). In such cases, the influence of problem difficulty on user problem selection becomes more pronounced. On the other hand, on the AIZU platform, users can submit problems at any time, and over time, even challenging problems receive more submissions, weakening the influence of difficulty on user problem selection. Lastly, we retained only the data from official competitions on AtCoder, as after the official competition ends, users can still participate in “Virtual Contests”. Participants in “Virtual Contests” have access to view other users' submitted code, which could potentially affect the accuracy of difficulty assessment. Therefore, in this study, we only kept the submission data from official AtCoder competitions. After preprocessing, the dataset consisted of 4,528,371 submission records. These preprocessing steps help us reduce the noise and heterogeneity in the data, and ensure the validity and reliability of our analysis.

### 3.4 Evaluating problem difficulty and examinee ability using the Rasch model

Item Response Theory (IRT) is a classical cognitive diagnostic method [20], [21] that allows for the assessment of item difficulty based on students' answer data. In this study, we employed the Rasch model, a well-known IRT model, to evaluate the difficulty parameters of the items using the user submission data from the OJ platform in the Project_CodeNet dataset. The Rasch model is a simple and effective way to estimate the difficulty and ability parameters based on binary outcomes, and it has been widely used in educational measurement and assessment. Difficulty parameters reflect the level of difficulty of the items, and hence, in our subsequent research, we used these parameters to stand for problem difficulty. The Rasch model [22] is widely used in practical applications due to its simplicity and usability for evaluating item difficulty in assessments, questionnaires, and other measurement tools. To compute the difficulty parameters of the problems using the Rasch model, the following steps were followed:

(1) For each programming problem, calculate the average acceptance rate of all submissions as the probability of the answer being correct for that problem.

In this study, the probability of the answer being correct is denoted as *p*. To compute this probability, we use the average acceptance rate of all submissions for a given problem as the probability of the answer being correct for that problem. The Rasch model assumes binary results, i.e., “correct” or “incorrect”. In the AtCoder data collected from Project_CodeNet, the number of test cases used in each submission is not provided, and only the submissions marked as “Accepted” receive a score, while other states such as “Wrong Answer” or “Compile Error” do not receive a score and are penalized. Therefore, all submission states in AtCoder can be categorized as either “Accepted” (solution correct) or “Unaccepted” (solution incorrect), which satisfies the assumption of the Rasch model. Hence, the average acceptance rate of all submissions represents the probability *p* of the answer being correct for a given problem.

(2) By utilizing the probability of the answer being correct, calculated using the Rasch model in IRT, we can determine the difficulty parameter for each programming problem.

The difficulty parameter, denoted as *diff* in this study, is computed by the probability of the answer being correct (*p*). The calculation of *diff* can be determined using Equation (1).

(1) $$\mathrm{diff}=\ln\left(\frac{1-p}{p}\right)$$

in IRT, the difficulty parameter is a real number, but it is typically constrained to a range of -2 to 2 [23]. This range limitation is imposed because the difficulty parameter reflects the level of difficulty of a task, and values outside this range may result in inconsistencies between the perceived difficulty and the actual difficulty of the task. In this study, the range of the difficulty parameter (*diff*) is restricted to -2 to 2. Any difficulty value exceeding 2 or falling below -2 is truncated to 2 or -2, respectively. A difficulty value closer to -2 indicates a lower difficulty level and higher likelihood of being answered correctly, while a value closer to 2 implies a higher difficulty level and lower probability of correct answers. The difficulty values obtained through Equation (1) represent the relative difficulty of a problem within its respective competition and cannot be directly compared across different competitions.

IRT can be used not only to evaluate the difficulty of problems but also to estimate the ability levels of individuals based on their submission data. In this study, the specific steps for assessing the ability levels of participants using the Rasch model are as follows:

(1) Calculate the acceptance rate for each user's participation in programming competitions and compute the average acceptance rate as the user's correctness rate, denoted as *cr*.

(2) Utilize the user's correctness rate (*cr*) and apply the Rasch model in IRT to calculate the ability value for each user.

The correctness rate (*cr*) is transformed logarithmically to compute the ability value, referred to as *ability* in this study. The *ability* can be calculated using Equation (2).

(2) $$ability=\ln\left(\frac{cr}{1-cr}\right)$$

the range of ability values in this study is constrained within -2 to 2. Any value exceeding 2 or falling below -2 is truncated to 2 or -2, respectively. A lower ability value indicates a lower level of proficiency, while a higher ability value suggests a higher level of proficiency for the user.

### 3.5 Data analysis methods

When investigating the influence of problem difficulty on submission behavior, the focus of the study is on the data related to the problems. Each problem is associated with a specific competition. Table 1 presents an example of problem data. In the table, “Submissions(%)” represents the proportion of attempts made on a particular problem compared to the total number of attempts on all problems in the competition. “Participants(%)” indicates the proportion of users who participated in answering the problem relative to the total number of users in the competition.

**Table 1:** An example of problem data.

Problem ID	Submissions (%)	Participants (%)	Difficulty
p02534	22.723	83.699	-1.636

Due to each problem being associated with a specific competition, it is not meaningful to discuss the numerical differences in submission counts and participant counts between problems when the competitions are different. Therefore, the submission counts and participant counts of problems are represented as percentages to reflect the submission situation within their respective competitions. In order to investigate the influence of problem difficulty on submission situation, this study divides all problems into four groups based on difficulty. The submission situation of problems within the same difficulty group is aggregated across all competitions, and the average values are obtained to represent the submission situation of different difficulty levels. Difficulty grouping is done using a sliding window approach, with a difficulty value range of [-2, 2], a fixed window size of 1.15, and a sliding distance of 0.95. Each group overlaps with a portion of size 0.2 with the previous group. The choice of a 1.15 window size and 0.95 sliding distance ensures that the four groups are of equal size, and the consistent sliding distance maintains uniformity in the movement of the sliding window. This grouping method prevents two problems with very close difficulty values (e.g., 0.999 and 1.001) from being assigned to different groups. If two problems are not in the same group, it guarantees that their difficulty values are not excessively close. The information after grouping is presented in Table 3.

When studying the factors that influence the answer sequence of users, the research focuses on the answer sequence and difficulty sequence of users. Each problem in the competition is assigned an unique index starting from 1. The answer sequence is a sequence of problem indices representing the order in which the user answers the problems in the competition. The difficulty sequence is a sequence of relative difficulty values of the problems answered by the user in the competition (see section 3.3 for the calculation of difficulty values). When an user submits a problem for the first time, the index and difficulty value of that problem are recorded in the answer sequence and difficulty sequence, respectively. After participating in the competition, user-related information, including user ID, competition ID, answer sequence, difficulty sequence, and the length of the answer sequence, is stored in the dataset as competition records. Table 2 presents examples of competition records. The “Length” column represents the length of the answer sequence, indicating the number of problems the user submitted in this competition. The “Answer Sequence” column contains the answer sequence, and the “Difficulty Sequence” column contains the corresponding difficulty sequence.

**Table 2:** Examples of user competition record.

User ID	Competition ID	Length	Answer sequence	Difficulty sequence
u508729896	14	4	1, 2, 3, 4	-1.325, 0.185, -0.852, 0.128
u018438536	286	6	1, 2, 3, 4, 5, 6	-1.593, 0.876, 1.020, 1.696, 0.990, 1.380
u168578024	119	6	5, 4, 6, 1, 2, 3	-0.768, 0.298, 0.112, -1.636, -1.067, -0.959

To ensure the accuracy of the research results, we processed the competition record data. Firstly, competition records with an answer sequence length of less than 3 were excluded. This was done to eliminate cases with excessively short answer sequences, as a length of 1 would not allow us to determine if the user's answers followed any specific order. Similarly, with a length of 2, the user's answers would always be in order, making it difficult to assess whether this order was influenced by the problem or difficulty sequence. Secondly, in the dataset, 94.58% of the competitions included at least 4 problems, and 96.89% of the competition records came from competitions with 4 or more problems. To reduce dataset heterogeneity, this study further excluded competition records with fewer than 4 problems. After processing, the dataset contained 514,128 competition records, corresponding to 514,128 answer sequences and difficulty sequences.

To investigate whether the order of user answer is influenced by the arrangement of problems or their difficulty, we computed the lengths of all continuously increasing or decreasing subsequences within the user's answer sequence (or difficulty sequence). Among these subsequences, the longest length is defined as the maximum ordered length in this study. Thus, the sequence's orderliness can be determined using the following definition:

• **Orderliness of the sequence**: Let the length of the sequence be denoted as *len_s*, and the maximum ordered length of the sequence as *len_o*. When *len_s*>3, if *len_o*>*len_s*/2, the sequence is considered ordered; otherwise, it is considered unordered. When *len_s* = 3, if *len_o* = *len_s*, the sequence is considered ordered; otherwise, it is considered unordered. This definition applies to both answer sequences and difficulty sequences in calculating their orderliness.

When studying whether the arrangement of problem difficulty affects user answer outcomes, the focus is on the difficulty ordering of problems in a competition and the user's answer outcomes. User answer outcomes refer to the user's performance in the competition, measured in terms of acceptance rate. The acceptance rate is calculated as the ratio of the number of correctly answered problems to the total number of problems in the competition. Regarding the difficulty arrangement of problems in a competition, there are three possible scenarios:

(1) Easy problems first, followed by difficult problems. In this case, the difficulty of the problems in the competition increases progressively. In this study, this arrangement is referred to as “easy to difficult”.

(2) Difficult problems first, followed by easy problems. In this case, the difficulty of the problems in the competition decreases progressively. This arrangement is referred to as “difficult to easy”.

(3) No apparent pattern or characteristic in the difficulty progression throughout the competition. In this case, the difficulty of the problems in the competition does not exhibit a clear trend. This arrangement is referred to as “no characteristic”.

Based on this, the trend of difficulty progression from the first problem to the last problem can be calculated to determine the difficulty arrangement of the problems in the competition. Specifically, all the problem difficulties in the competition are treated as a sequential series of numbers. By performing linear regression on this series and calculating the slope of the regression equation, the trend of difficulty progression can be determined. To more accurately distinguish the “no characteristic” scenario from the other two scenarios with clear trends, a threshold of 0.05 is set in this study. Specifically, when the slope value is greater than or equal to 0.05, it indicates an increasing trend in difficulty, suggesting that the earlier problems in the competition have relatively lower difficulty values, while the later problems have relatively higher difficulty values, i.e., “easy to difficult”. When the slope value is less than or equal to -0.05, it indicates that the earlier problems have relatively higher difficulty values, while the later problems have relatively lower difficulty values, i.e., “difficult to easy”. When the absolute value of the slope is less than 0.05, it represents no clear characteristic in the arrangement of problems in the competition, i.e., “no characteristic”.

## 4 Results and discussion

### 4.1 Analysis of the influence of problem difficulty on submission situation

#### 4.1.1 The influence of problem difficulty on submission situation

Table 3 is the problem difficulty grouping statistics table, which displays the submission status after categorizing the problems in each competition into four groups based on difficulty. The “Group” column indicates the grouping, in which the difficulty of problems increases across the groups, with Group 1 having the lowest difficulty and Group 4 having the highest difficulty. The “Difficulty” column indicates the upper and lower limits of the difficulty values within each group. The “problem Number” column indicates the number of problems in each group. For a given problem, the “Accepted(%)” column represents the average acceptance rate of all submissions for that problem. This grouping method helps us compare the submission situation of different difficulty levels, and investigate how problem difficulty influences students' learning behavior on OJ platforms.

**Table 3:** Problem difficulty grouping statistics table.

Group	1	2	3	4
Difficulty	[-2, -0.85]	[-1.05, 0.1]	[-0.1, 1.05]	[0.85, 2]
Problem number	332	436	551	444
Accepted(%)	77.929	61.393	38.537	19.729

**Figure 1:**
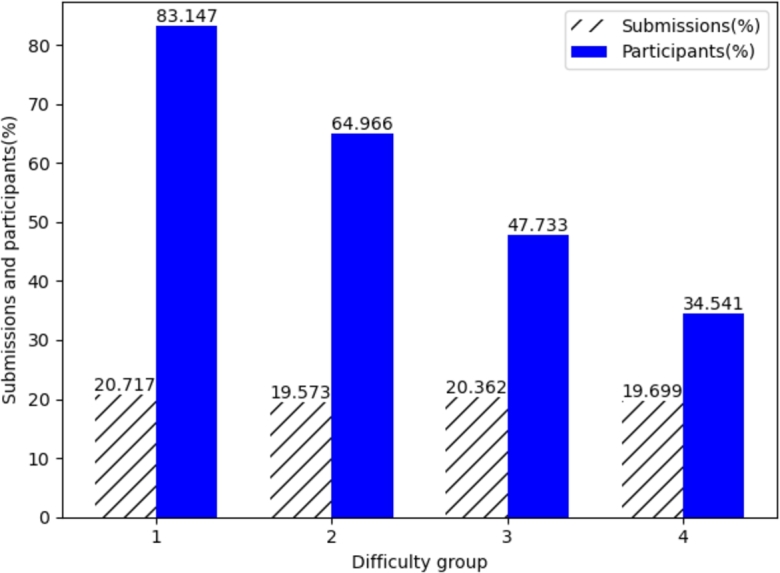
The influence of problem difficulty on submissions and participants.

From Table 3, it can be observed that as the difficulty increases, the average acceptance rate of the problems decreases. This aligns with the relationship between difficulty and acceptance rate. Fig. 1 illustrates the influence of difficulty on the number of submissions and participants. The “submissions(%)” represents the average proportion of submissions for all problems within a group in their respective competitions, while “participants(%)” represents the average proportion of participants who submitted solutions for all problems within a group in their respective competitions. The dashed line in the figure represents the trend line obtained through linear regression, illustrating the trend of the data. The influence of problem difficulty on submission patterns is significant. In Fig. 1, it can be observed that as the problem difficulty increases, the number of submissions show a slight decline, although this trend is not pronounced and can be disregarded. However, the number of participants significantly decreases with an increase in problem difficulty.

The decrease in the number of participants may result from the increasing difficulty that discourages many users from attempting these challenging problems. Additionally, considering the context of the data being sourced from OJ competitions, participants aim to earn more points within a limited timeframe. Therefore, for excessively difficult problems, they may choose to abandon them and focus on relatively simpler problems where they can score points. However, the number of submissions does not decrease with the reduction in participants. Higher difficulty groups contribute the same number of submissions as lower difficulty groups with a larger number of participants. This indicates that more difficult problems have a higher average number of attempts per person. This phenomenon may be due to the fact that more challenging problems require more thinking and attempting to arrive at the correct solution. The decline in the average acceptance rate in Table 3 also supports this observation, as higher difficulty problems are more difficult to be accepted, leading users to make more debugging and submission attempts.

These findings provide valuable insights for developers of OJ systems. When designing and developing educational software and online learning platforms, the influence of problem difficulty on users should be taken into consideration to avoid discouraging users by setting the difficulty level too high. Additionally, more challenging problems require more thinking and experimentation. This implies that in non-competitive scenarios, designers should provide users with sufficient time and opportunities to attempt problem-solving, rather than emphasizing rapid task completion or restricting the number of attempts. For excessively difficult problems, students need to have a solid understanding of fundamental concepts, employ effective methods and strategies, and make multiple attempts to tackle the problems. Therefore, in programming education, teachers can offer appropriate guidance and support to students when they encounter challenging tasks, helping them solve problems more effectively and overcome their fear of difficulties. Similarly, OJ platforms can provide suitable assistance and hints to users when they face difficult problems, boosting their confidence.

#### 4.1.2 Analysis of the influence of problem difficulty on submission patterns: Spearman correlation approach

In examining the influence of problem difficulty on submission situation, this section employed Spearman correlation Analysis to calculate the correlation coefficients between the difficulty level and the number of submissions, as well as the difficulty level and the number of participants. The analysis results are presented in Table 4 and Table 5, with p-values indicated in parentheses. In these tables, ***,**,* represent significance levels at 1%, 5%, and 10% respectively. Table 4 displays the correlation coefficients between the difficulty level and the proportion of submissions.

**Table 4:** Difficulty and submissions correlation coefficient table.

	Difficulty	Submissions
Difficulty	1 (0.000***)	-0.084 (0.001***)
Submissions	-0.084 (0.001***)	1 (0.000***)

Note: ***,**,* represent significance levels at 1%, 5%, and 10% respectively.

**Table 5:** Difficulty and participants correlation coefficient table.

	Difficulty	Participants
Difficulty	1 (0.000***)	-0.533 (0.000***)
Participants	-0.533 (0.000***)	1 (0.000***)

Note: ***,**,* represent significance levels at 1%, 5%, and 10% respectively.

The Spearman correlation coefficient ranges from -1 to 1, where -1 indicates a perfect negative correlation, 1 indicates a perfect positive correlation, and 0 indicates no correlation. In the analysis results presented in Table 4, the correlation coefficient between difficulty and the proportion of submissions is -0.084, suggesting a slight negative correlation between difficulty and submission proportion. Although the p-value is less than 0.05, indicating statistical significance, the relationship is not strong.

Table 5 displays the correlation coefficients between difficulty and the proportion of participants. In the analysis results of Table 5, the correlation coefficient between difficulty and the proportion of participants is -0.533, indicating a strong negative correlation between difficulty and participant proportion. This means that as the difficulty of the problems increases, the proportion of participants decreases. Moreover, the p-value is less than 0.05, indicating that this correlation is not due to randomness but is statistically significant. This correlation is indeed present and is not caused by sampling errors or random factors.

The results of the Spearman correlation analysis confirm the relationship between problem difficulty and submission situation. Specifically, there is a slight negative correlation between problem difficulty and the number of submissions, although this relationship is very weak and can be considered negligible. On the other hand, there is a strong and highly significant negative correlation between problem difficulty and the number of participants. This correlation is statistically meaningful and further supports the findings in section 4.1.1, indicating that problem difficulty plays a significant role in submission situation.

### 4.2 Analysis of factors influencing user answer order

**Figure 2:**
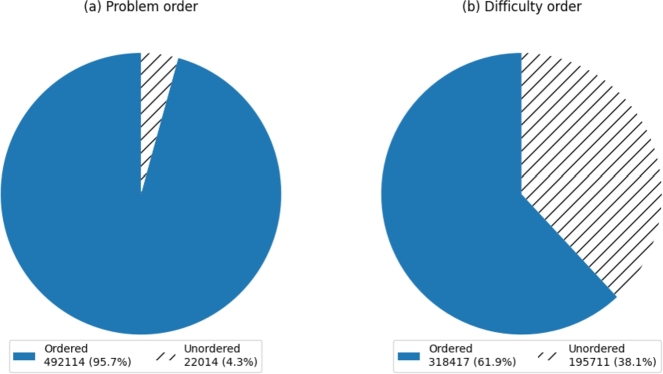
Proportion of users' answer problems in order or disorder according to problem order and difficulty order.

In this section, the 514,128 competition records were processed according to the definition of orderliness of the sequence in section 3.4. Fig. 2 illustrates the proportions of ordered and unordered data obtained after processing. Subfigure (a) presents the results based on the problem order, while subfigure (b) presents the results based on the difficulty order. From subfigure (a), it can be observed that in 492,114 records (95.72% of the total), users' answer order were ordered according to the arrangement of the problems. In the remaining 22,014 records (4.28% of the total), users' answer order were not ordered according to the problem arrangement. From subfigure (b), it can be observed that in 318,417 records (61.93% of the total), users' answer order were ordered according to the difficulty order of the problems. In the remaining 195,711 records (38.07% of the total), users' answer order were not ordered according to the difficulty order of the problems.

Due to the non-normal distribution of the data, this study employed the one-sample Wilcoxon signed-rank test to perform a statistical analysis, and the results are presented in Table 6. The results of the one-sample Wilcoxon signed-rank test indicate that the large sample size, high z-values, and statistically significant p-values demonstrate a strong level of orderliness in the data. This suggests that the majority of competition records exhibit a tendency towards orderliness. The test results provide evidence that users' answer sequences possess a certain degree of orderliness rather than being completely random. It implies that users are more likely to answer problems in accordance with the order of the problems or the order of difficulty, rather than choosing them randomly and in a disorderly manner.

**Table 6:** One-Sample Wilcoxon Signed-Rank Test.

Name	Test statistic	Sample size	Standard deviation	Z	P
Problem order	0.957	514128	0.202	584.982	0.000***
Difficulty order	0.619	514128	0.486	150.191	0.000***

Note: ***,**,* represent significance levels at 1%, 5%, and 10% respectively.

These findings indicate that both the order of problems and their respective difficulty levels exert a significant influence on users' answer sequences. Furthermore, the order of problems has a more pronounced impact on users' answer sequences. In the course of addressing problems, the predominant pattern observed involves users attempting to answer problems sequentially. In instances where an insurmountable challenge is encountered, a majority of users opt to bypass such challenging problems temporarily, instead choosing to tackle those problems they can readily solve. This is evident from the higher z-value observed in the test for problem order. The magnitude of the z-value reflects the degree of orderliness in the data, with a larger z-value indicating a stronger degree of order. In the test for problem order, the z-value is 584.982, while in the test for difficulty order, the z-value is 150.191. The higher z-value in the test for problem order suggests a stronger influence of problem order on the user answer sequence. The vast majority of users opt for a sequential approach in answering questions. This is likely due to the fact that systematically completing tasks can aid them in tracking their progress more efficiently, preventing any confusion during reviews. Furthermore, when users encounter challenging questions, they often choose to address the simpler ones first and later circle back to the more demanding questions. Such a strategy might be rooted in considerations of efficiency, aiming to first secure easily attainable scores and subsequently allocate more time and effort to tackle the more challenging problems.

### 4.3 Analysis of the influence of problems difficulty arrangement order on users' answering outcomes

#### 4.3.1 Classification of user ability level

In order to investigate the influence of the arrangement of problem difficulty on user performance, we divided users into three categories based on their ability values using K-means clustering. Categories 1, 2, and 3 represent users with low, medium, and high ability levels respectively. Utilizing K-means clustering for grouping or classifying data is a common practice [24]. Table 7 presents the results of the field differences analysis for the K-means clustering. In Table 7, Low-level(18191) indicates that 18191 users were classified as low ability level users. Similarly, Medium-level(43942) and High-level(20453) represent 43942 users and 20453 users respectively, identified as medium ability level and high ability level users.

**Table 7:** Table of field differences analysis.

	Low-level(18191)	Medium-level(43942)	High-level(20453)	F	P
	Mean±Standard deviation	Mean±Standard deviation	Mean±Standard deviation		
Ability	-1.476±0.362	-0.278±0.313	0.770±0.462	181297.700	0.000***

Note: ***,**,* represent significance levels at 1%, 5%, and 10% respectively.

The F-test results showed significant differences in the means of the fields between different clusters (P<0.05), indicating that the clustering results are statistically meaningful and represent different user groups. This demonstrates the appropriateness of using the K-means method for user classification. The silhouette coefficient is 0.553, and the DBI index is 0.57, indicating a high quality of the classification results and moderate data distinctiveness between clusters, signifying a good classification. The CH index is relatively high, at 181297.7, indicating a good quality of the clustering results. Multiple runs of the K-means algorithm yielded stable classification results. Therefore, the classification of users based on their ability values using K-means clustering is statistically meaningful, with a good-quality classification and reliable stability.

#### 4.3.2 Analysis of differences in users' answering outcomes under different difficulty arrangement order

Table 8 presents the results of the Mann-Whitney U Test, which shows the analysis of differences in acceptance rates among different levels of users under different difficulty ordering of problems. In the Table 8, group ACR_A (ACR i.e., acceptance rate, A i.e., all) represents the group without distinguishing users' ability levels, while group ACR_H (H i.e., high), ACR_M (M i.e., median), and ACR_L (L i.e., low) represent the groups where users' ability levels are labeled as high, medium, and low, respectively. In this study, DAO means “difficulty arrangement order”. In the DAO column, 1 represents “easy to difficult” data, -1 represents “difficult to easy” data, and 0 represents “no characteristic” data. When DAO is 1 and 0, it indicates the Mann-Whitney U Test Analysis results between the difficulty ordering of problems “easy to difficult” and “no characteristic”. Similarly, 1 and -1 represent “easy to difficult” and “difficult to easy”, while 0 and -1 represent “no characteristic” and “difficult to easy”.

**Table 8:** Differences in users' answer results under different difficulty arrangement order: results of Mann-Whitney U Test Analysis.

Group	DAO		Sample size		Median		Test statistic	P	Median difference	Cohen's d value
ACR_A	1	0	244	22	0.545	0.308	4410.0	0.000***	0.237	1.185
	1	-1	244	29	0.545	0.309	5872.5	0.000***	0.236	1.304
	0	-1	22	29	0.308	0.309	304.5	0.783	0.001	0.088

ACR_H	1	0	244	22	0.666	0.359	4427.5	0.000***	0.306	1.281
	1	-1	244	29	0.666	0.372	5848.5	0.000***	0.294	1.324
	0	-1	22	29	0.359	0.372	308.0	0.834	0.013	0.038

ACR_M	1	0	243	21	0.479	0.224	4186.0	0.000***	0.255	1.223
	1	-1	243	29	0.479	0.234	5895.0	0.000***	0.245	1.349
	0	-1	21	29	0.224	0.234	318.5	0.783	0.010	0.065

ACR_L	1	0	240	20	0.227	0.118	3800.5	0.000***	0.109	0.866
	1	-1	240	28	0.227	0.114	5925.0	0.000***	0.113	1.647
	0	-1	20	28	0.118	0.114	243.0	0.439	0.004	0.433

Note: ***,**,* represent significance levels at 1%, 5%, and 10% respectively; DAO: difficulty arrangement order.

For the “difficult to easy” and “no characteristic” data, the Cohen's d values for ACR_A, ACR_H, and ACR_M are all less than 0.2, indicating very small effect sizes. The Cohen's d value for ACR_L is 0.433, approaching 0.5, indicating a moderate effect size. However, regardless of whether users' ability levels are distinguished or not, the p-values for the “difficult to easy” and “no characteristic” data are greater than 0.05, indicating non-significant results. In other words, for users at any level, there is no significant difference in acceptance rates between the two difficulty ordering sequences of “difficult to easy” and “no characteristic”.

For ACR_A, ACR_H, ACR_M, and ACR_L in both “easy to difficult” and “no characteristic” groups, the Cohen's d values exceed 0.8, indicating that the magnitude of differences is very large in all cases. Moreover, the p-values are less than 0.05, reaching a significant level. This implies a significant difference in acceptance rates between “easy to difficult” and “no characteristic” for users of any level. Furthermore, this difference has a consistent direction: when the difficulty arrangement order is “easy to difficult”, the acceptance rate is significantly higher than the acceptance rate when the ordering sequence has “no characteristic”.

Similar to the “easy to difficult” and “no characteristic” data, there is also a significant magnitude of difference between the “easy to difficult” and “difficult to easy” data: the Cohen's d values for all groups exceed 1.3. The p-values are less than 0.05, which indicate the statistical significance results. Therefore, for users at any level, there is a significant difference in acceptance rates between the two difficulty arrangement order of “difficult to easy” and “easy to difficult”: when the difficulty arrangement order is “easy to difficult”, the user acceptance rate is significantly higher than the acceptance rate when the difficulty arrangement order is “difficult to easy”.

Furthermore, through comparisons, it can be observed that the difference in acceptance rates between the “easy to difficult” data and the “difficult to easy” data is even larger compared to the differences between the “easy to difficult” data and the “no characteristic” data. These results indicate that when the difficulty arrangement order is “easy to difficult”, the user's acceptance rate is the highest, significantly differing from other difficulty arrangement order with a substantial effect size. When the difficulty ordering sequence is “difficult to easy” or “no characteristic”, the user's acceptance rate is significantly lower compared to the “easy to difficult” scenario. However, there is no significant difference in the acceptance rate between the “difficult to easy” data and the “no characteristic” data.

Based on the findings of RQ2, users tend to prefer answering problems in an orderly manner according to the sequence of the problems. The ordering of problem difficulty significantly affects users' answer sequences. Combining these findings can assist teachers in implementing targeted assessments of students' programming skills. Consequently, in the field of computer-assisted instruction and programming education, teachers can dynamically adjust the difficulty and sequence of problems based on students' abilities and progress to accommodate their learning needs and provide appropriate challenges. This personalized learning path can enhance student engagement, learning effectiveness, and self-directed learning abilities. When designing programming exams, teachers can guide students by altering the ordering of problem difficulty, taking into account their tendency to answer in an orderly manner. Teachers may place easier problems at the beginning to stimulate students' enthusiasm and prevent premature abandonment or conduct targeted tests before important competitions or exams to boost students' confidence. Students should be cautious of the potential negative effects of their answering habits. If they encounter challenging problems early on, it is important to adjust their mindset promptly, avoiding misjudging the overall difficulty of the test based on the problem's position and consequently experiencing psychological pressure leading to poor performance.

### 4.4 The influence of different difficulty arrangement order on cognitive load

#### 4.4.1 Correlation between cognitive load and user answer result

Cognitive load [9], [25] refers to the amount of information or information processing required by an individual during a cognitive activity. In the context of programming competitions, it can be understood as the mental resources or information processing involved in completing a certain number of tasks of varying difficulty. When the difficulty of the tasks is ordered from easy to difficult, there are significant differences in users' answer outcomes compared to other ordering sequences, which may be attributed to differences in cognitive load.

Cognitive load can affect concentration, clarity of thought, and subsequently influence the effectiveness of answer. Based on the findings of RQ2, users are likely to answer problems in the order presented. Different difficulty arrangement order can result in variations in information processing demands and, consequently, differences in cognitive load. Cognitive load influences cognitive resources and thinking processes, and an increased cognitive load can lead to divided attention, increased difficulty in judgment, and an influence on answer outcomes. Therefore, we can make a reasonable assumption that different difficulty ordering sequences lead to differences in cognitive load, which in turn result in significant variations in answer outcomes.

#### 4.4.2 Assessments of cognitive load: based on Project_CodeNet dataset

Cognitive load is typically assessed through biological indicators (e.g., eye movement frequency and heart rate variability), self-reports (e.g., questionnaires), or behavioral measures [26]. To measure cognitive load more accurately, this study collected three indicators from users' submission information that are highly correlated with cognitive load: time interval (the duration between users' submissions for different problems), problem switching number (the number of times when user switch problems without solving the previous one), and error rate in submissions. Increased cognitive load can result in attentional distractions and decreased cognitive control [27]. Therefore, by assessing users' levels of attentional distractions (e.g., problem switching number, time interval) and cognitive control abilities (e.g., error rate), changes in cognitive load can be evaluated. Behavioral measures such as accuracy (e.g., error rate) and response time also provide reliable assessments of cognitive load [28].

The time interval between users' submissions for different problems reflects the time consumption for thinking about the next problem after enduring cognitive load from the previous one. The longer the consumption, the greater the cognitive load. When users switch problems without being accepted the previous one, it indicates that the previous problem was difficult for the user. The more frequent these switches occur, the less the user is able to concentrate. The level of attentional concentration is a reflection of cognitive load, where decreased concentration implies a higher cognitive load. An increase in submission error rate suggests that individuals face certain obstacles or deficiencies in controlling and supervising cognitive problems. With an increase in cognitive load, individuals need to allocate more attentional resources to complete the cognitive problems, which can affect the control and supervision of problem details and increase the likelihood of submission errors. Therefore, to some extent, a higher error rate can reflect a higher cognitive load experienced by individuals. This study measures users' cognitive load using the three indicators mentioned. Lastly, the study performs min-max normalization on these three indicators and calculates their mean as the evaluation value for cognitive load in this research.

Specifically, the statistical methods for the three indicators are as follows:

• Time interval: If the current submission is for a different problem than the previous one, calculate the time interval and take the maximum value among all intervals. A larger value indicates a greater cognitive load.

• Problem switching number: Calculate the number of times when user switches problems without solving the previous one. A higher value indicates a greater cognitive load.

• Error rate: Let the number of submissions is *s_num*, the number of problems being accepted is *ac_num*, and the error rate is *er*. The error rate can be calculated using Equation (3). A higher value indicates a greater cognitive load.

(3) $$er=\left(\frac{s\_num-ac\_num}{s\_num}\right)$$

#### 4.4.3 Analysis of differences in cognitive load under different difficulty arrangement order

To investigate whether different difficulty arrangement order of problems leads to differences in cognitive load experienced by users, this study conducted a differential analysis of cognitive load indicators under different difficulty arrangement order. The results of the analysis are presented in Table 9. In the Table 9, group CL_A (CL i.e., cognitive load, A i.e., all) represents the group where user ability levels are not differentiated, while group CL_H (H i.e., high), CL_M (M i.e., median), and CL_L (L i.e., low) represent the groups where user ability levels are labeled as high, median, and low, respectively. The meaning of DAO is the same as in Table 8.

**Table 9:** Differences in cognitive load: results of Mann-Whitney U Test Analysis.

Group	DAO		Sample size		Median		Test statistic	P	Median difference	Cohen's d value
CL_A	1	0	593471	18133	0.210	0.259	4346394076.5	0.000***	0.049	0.349
	1	-1	593471	19790	0.210	0.296	3679881338.5	0.000***	0.086	0.669
	0	-1	18133	19790	0.259	0.296	150621580.5	0.000***	0.037	0.295

CL_H	1	0	252356	9517	0.196	0.256	914322721.5	0.000***	0.059	0.444
	1	-1	252356	11878	0.196	0.287	944751147.0	0.000***	0.09	0.671
	0	-1	9517	11878	0.256	0.287	63104837.0	0.000***	0.031	0.207

CL_M	1	0	316580	7944	0.216	0.258	1057800147.5	0.000***	0.043	0.279
	1	-1	316580	7403	0.216	0.305	690151501.5	0.000***	0.089	0.733
	0	-1	7944	7403	0.258	0.305	22893713.0	0.000***	0.047	0.415

CL_L	1	0	24535	672	0.275	0.301	7161195.5	0.000***	0.027	0.204
	1	-1	24535	509	0.275	0.360	3203731.5	0.000***	0.085	0.779
	0	-1	672	509	0.301	0.360	227832.0	0.000***	0.059	0.591

Note: ***,**,* represent significance levels at 1%, 5%, and 10% respectively; DAO: difficulty arrangement order.

The results indicate that there are significant differences (P<0.05) in cognitive load indicators when the difficulty arrangement order of problems varies. When analyzing the data with difficulty arrangement order of “easy to difficult” and “no characteristic”, high-level users show a higher magnitude of cognitive load difference compared to other users. The Cohen's d value for high-level users reaches 0.444, which is close to 0.5, indicating a moderate effect. For other ability levels, the Cohen's d values are around 0.2, indicating smaller effects. This could be attributed to the confidence-boosting effect of the “easy to difficult” difficulty arrangement order, which is more effective for high-level users, significantly reducing their cognitive load.

In the “difficult to easy” and “no characteristic” data, the Cohen's d value for the high-level user group is 0.207, indicating a relatively small effect size of cognitive load difference. The Cohen's d value for the median-level user group is 0.415, close to 0.5, indicating a moderate-to-low effect size. Only the low-level user group has a Cohen's d value exceeding 0.5, indicating a moderate effect size. In this group, there are noticeable differences in the magnitude of cognitive load across users at different ability levels.

Among all the data group, the largest difference in magnitude is observed between the “easy to difficult” and “difficult to easy” group. For users at any proficiency level, the Cohen's d value exceeds 0.5, approaching 0.8, indicating a large effect size. Specifically, the high-level user group shows the smallest difference with a magnitude of 0.671, while the low-level user group exhibits the largest difference with a magnitude of 0.779. This suggests that regardless of learners' ability levels, the “difficult to easy” difficulty arrangement order has a significantly higher influence on cognitive load compared to the “easy to difficult” difficulty arrangement order, greatly influencing the learning experience and outcomes.

As a result, we can observe that when the difficulty order of the problems is “easy to difficult”, users experience the lowest cognitive load, followed by the “no characteristic” order. When the difficulty order is “difficult to easy” the cognitive load is the highest, and the influence is more pronounced for users with lower ability levels. These findings provide insights for computer-assisted instruction, where relevant educational systems can consider these conclusions when designing material difficulty order and learning strategies tailored to learners of different abilities. In programming education, instructors should adopt appropriate task difficulty ordering methods for students with different abilities to reduce cognitive load and improve learning outcomes. In test design, teachers can influence students' cognitive load during the answering process by changing the order of problem difficulty, to some extent, affecting students' performance, boosting confidence, or preventing complacency.

### 4.5 Implications of OJ's application for the overall learning process

When students are in the initial stages of learning, it is desirable for them to experience a sense of achievement promptly. Adjusting the difficulty arrangement order of problems to “easy to difficult” facilitates improved outcomes in problem-solving, thereby enjoying the learning process more. As students utilize OJ platforms for programming practice and skill enhancement, the difficulty level can be incrementally raised. Students can be provided with some challenges by setting some challenging problems. At the examination phase, differentiation in evaluating students' learning outcomes becomes crucial. Altering the problem sequence to “difficult to easy” or “no characteristic” can more effectively discern between students' ability levels.

Additionally, teachers can make more specific adjustments based on their objectives. For instance, teachers can adjust the difficulty arrangement order of problems to help students achieve better results, thereby boosting their confidence ahead of significant examinations. They can also adjust the difficulty arrangement order to negatively impact students' performance in routine practice-oriented tests to a certain degree, preventing students from becoming overly proud.

## 5 Conclusions and limitations

In this study, we explored how the difficulty level and the order of programming problems on OJ platforms affect students' learning behavior, performance, and cognitive load. This study's findings offer pivotal insights for programming education and the conceptualization and enhancement of OJ systems and other digital instructional tools. Statistical analyses were deployed to scrutinize the impact of problem difficulty on submission situations, determinants that sway users' problem-solving sequence, and the nexus between difficult arrangement orders of problems with answer outcomes and cognitive load. The research data were sourced from Project_CodeNet. The outcomes revealed a pronounced influence of problem difficulty on submission situations. In contests, problems of heightened difficulty garnered fewer participants in contrast to simpler problems which drew a larger crowd. Nonetheless, the submission count remained relatively consistent between challenging and simpler problems, signifying more attempts were necessitated for the formidable problems. Such revelations can guide OJ system developers in crafting educational software and online educational platforms, prompting an acknowledgment of the bearing of problem difficulty on users. In both pedagogical and non-competitive contexts, educators and OJ system architects must ensure ample time and avenues for learners to tackle problem-solving, furnishing pertinent assistance during challenging phases.

The sequence in which users tackle problems is shaped by both the order in which problems are presented and their difficulty. During competitions, participants frequently address problems sequentially. However, when confronted with particularly challenging problems beyond their grasp, they may deliberately bypass these and prioritize simpler ones. Notably, this investigation determined that the difficult arrangement orders of problems markedly affect users' problem-solving outcomes. When problems are arranged in ascending order of difficulty (“easy to difficult”), the users' acceptance rates are significantly higher compared to the arrangement orders of “no characteristic” or “difficult to easy”. These findings can assist teachers in implementing targeted assessments of students' programming skills, enabling them to adjust the arrangement orders of problem difficulty based on students' abilities and progress, in order to meet their learning needs and provide appropriate challenges.

This study comprehensively evaluated behavioral indicators such as “time interval”, “problem switching number”, and “error rate” in programming competitions to analyze and study the cognitive load under different arrangements of problem difficulty. The results indicate that users of different ability levels experience varying degrees of cognitive load under different arrangements of problem difficulty. Significant differences exist in the cognitive load experienced by users under the three different arrangements, with slight variations in the magnitude of the differences. Overall, when problems are arranged in ascending order of difficulty (“easy to difficult”), users experience the lowest cognitive load. When problems are arranged in descending order of difficulty (“difficult to easy”), the cognitive load is highest, especially for users with lower ability levels. The “no characteristic” random arrangement falls between the two. These findings provide insights for computer-assisted teaching, and educational practitioners can refer to these conclusions to develop teaching plans that balance the cognitive load of students in learning and exams by adjusting the arrangement orders of problem difficulty in exercises and assessments, thereby facilitating better programming learning outcomes.

The data for this study is derived from competitions, and currently, there is no source of data originating from educational contexts. Therefore, it's not possible to verify if the conclusions of this study are feasible in educational settings. Furthermore, the Project_CodeNet dataset only contains data on programming problems and lacks data on other types of problems. Consequently, the conclusions of this research are specifically for programming problems, and it's uncertain if they apply to other types of questions (such as objective questions or other types of subjective questions).

Due to the limitations of the dataset, this study did not consider factors such as users' knowledge background, geographic location, gender, coding proficiency, and the knowledge domains of the problems. In future research, it would be beneficial to explore these aspects. For example, investigating users' submission patterns across different knowledge domains to examine whether there are significant differences in mastery levels among users of varying ability, as well as studying the differences in coding styles among users of different ability levels.

Finally, the “Results and discussion” section discusses how the conclusions of this study can better guide teachers in using OJ to assist the overall learning process. However, whether these insights can truly be effective has not been explored through case studies, nor have relevant supporting literature been found. In future work, consideration will be given to conducting several related controlled experiments to verify whether these insights can genuinely take effect.

## CRediT authorship contribution statement

**Jinshui Wang:** Conceptualization, Formal analysis, Funding acquisition, Methodology, Writing – review & editing. **Pengchen Lin:** Data curation, Formal analysis, Investigation, Methodology, Software, Writing – original draft, Writing – review & editing. **Zhengyi Tang:** Conceptualization, Formal analysis, Methodology, Writing – review & editing. **Shuguang Chen:** Software, Writing – original draft.

## Declaration of Competing Interest

The authors declare that they have no known competing financial interests or personal relationships that could have appeared to influence the work reported in this paper.

## Data Availability

The data that support the findings of this study are available in Project_CodeNet: AtCoder official contest and derived data at https://zenodo.org/record/8141645, DOI https://doi.org/10.5281/zenodo.8141645. These data were derived from the following resources available in the public domain: - IBM/Project_CodeNet, https://github.com/IBM/Project_CodeNet. - IBM/Project_CodeNet: Initial release 1.0 (May 5, 2021), https://zenodo.org/record/4814770.
